# Challenging Case Report: Reevaluating autism diagnosis in a 7-year-old girl

**DOI:** 10.3389/fpsyt.2025.1701629

**Published:** 2026-02-09

**Authors:** Hagit Nagar-Shimoni, Noa Leibovitch, Efrat Zilbershot Fink, Yuval Cnaan, Yael Leitner

**Affiliations:** 1 Marot Autism Center, Child Development Institute, Dana-Dwek Children’s Hospital, Tel Aviv, Israel; 2 Max Stern Yezreel Valley Academic College, Psychology Department, Afula, Israel; 3 Child Developmental Unit, Meuhedet Health Services, Central District, Hod Hasharon, Israel; 4 Child Developmental Center, Clalit Health Services, Sharon-Shomron District, Kadima Zoran, Israel; 5 Clalit Health Services, Tel Aviv District, Tel Aviv, Israel; 6 Faculty of Medicine, Tel Aviv University, Tel Aviv, Israel

**Keywords:** a pediatric case, ADOS, Autism spectrum disorder (ASD), diagnostic challenges, girls, neurology, group observation

## Abstract

Diagnosing autism spectrum disorder (ASD) in verbally fluent, socially motivated children, particularly girls, remains challenging due to compensatory behaviors and masking. This case report follows a 7-year-old girl repeatedly referred to autism spectrum evaluation. Despite early developmental delays, attentional variability, and sensory sensitivities, initial community-based multidisciplinary assessment excluded ASD but did not resolve parental concerns. A second evaluation at the hospital-based developmental clinic included neurological and psychological assessments, revealing divergent impressions and prompting naturalistic peer-group observations. In these naturalistic settings, the girl demonstrated strong social interest, reciprocity, emotional expressiveness, and adaptability, with no repetitive behaviors or marked rigidity. Support was required for attention and frustration management, but overall social interaction quality was age-appropriate given her developmental profile. The final diagnosis ruled out ASD. The observed difficulties were instead attributed to below-average cognitive functioning, Attention Deficit Hyperactivity Disorder, language difficulties and immature socio-emotional development. This case highlights the value of integrating longitudinal, naturalistic peer-group observations with standardized tools to provide ecologically valid insights into adaptive functioning. Such methods are particularly useful in complex diagnostic cases and align with recent calls to refine procedures for girls with social difficulties. In the present case, accurate diagnostic clarification required sustained multidisciplinary teamwork, clear communication with the parents, and the incorporation of naturalistic peer-group observation alongside standardized assessment tools.

## 1 Introduction

Autism spectrum disorder (ASD) is a neurodevelopmental condition characterized by persistent deficits in social communication and social interaction across multiple contexts, along with restricted, repetitive patterns of behavior, interests, or activities (1). Diagnosing ASD in verbally fluent, socially curious children, especially girls, presents ongoing challenges for clinicians. Traditional diagnostic tools such as the Autism Diagnostic Observation Schedule, Second Edition (ADOS-2) (2) provide standardized criteria but do not always account for compensatory behaviors and developmental masking, which are more common among girls (3, 4). Notably, accumulating evidence indicates that standard diagnostic procedures may under-identify autism in girls and women, highlighting the need for assessment approaches that better capture female presentations and contextual variability (3, 5). Moreover, the ADOS-2 does not differentiate between the various sources that contribute to social communication difficulties. In many healthcare systems, including the Israeli public healthcare system, an ASD diagnosis provides access to extensive therapeutic, educational, and welfare resources, including multidisciplinary interventions and individualized educational accommodations. Misdiagnosis can lead to inappropriate interventions or delayed support for alternative conditions such as developmental delay, emotional or attentional disorders.

In the child’s health care system, the diagnostic process for ASD is guided by national Ministry of Health regulations and is based on a dual evaluation model, requiring independent assessments by both a physician and a licensed psychologist with specialized training in autism diagnosis. The diagnosis is grounded in DSM-5 criteria and integrates standardized assessment tools alongside clinical judgment, with formal recognition contingent upon concordance between medical and psychological evaluations. While this structured framework enhances diagnostic rigor, it may also give rise to differing emphases across evaluators, particularly when clinical impressions diverge from findings obtained through standardized instruments. In this context, complementary assessment settings—such as naturalistic and peer-based observations—may play a critical role in supporting differential diagnosis and clarifying complex developmental presentations.

This case report traces the diagnostic journey of a 7-year-old girl in the Israeli public healthcare system who was repeatedly referred to ASD evaluation. Over time, through individual assessments by medical and allied health professionals, two multidisciplinary evaluations, and naturalistic peer-group observations, the clinical impression evolved. Rather than ASD, the findings pointed to developmental delays accompanied by attentional and adaptive difficulties, language difficulties, below average IQ, and emotional immaturity. This case underscores the value of naturalistic observations, collaborative team interpretation, and context-sensitive assessment in navigating complex developmental profiles (6–9).

## 2 Case description

### 2.1 Developmental and medical history

At the time of referral to the hospital-based developmental clinic located in a metropolitan city, the child was 7 years and 6 months old and enrolled in a first-grade special education classroom within a mainstream school setting. She received in-class support from a teaching aide, speech therapist, and clinical psychologist. She is the second child in the family, with an older sister who is reportedly typically developing.

Developmental delays were evident from infancy: walking achieved after 18 months, and first words emerged around age two. All major milestones were delayed, and she attended municipal preschool at age three. There were no additional medical, developmental, or family factors identified that could account for her difficulties, beyond the documented history of ADHD, suspected intellectual disability, and a speech and language disorder.

Developmental history reviewed by the neurologist at the hospital-based developmental clinic showed that the child had undergone multiple evaluations over the years. At age 4, a psychological evaluation was conducted using the Wechsler Preschool and Primary Scale of Intelligence (WPPSI-III) (10), which indicated a full-scale IQ of 78, with a verbal IQ of 77, performance IQ of 94—revealing a significant discrepancy between verbal and nonverbal domains.

At age 5, prior to her referral to the hospital-based developmental clinic, she was evaluated by a neurologist, a psychiatrist, and a clinical dietitian. All of them recommended an autism spectrum evaluation. When she was 7 years old, a first multidisciplinary assessment was conducted in the community by a neurologist and a psychologist. This evaluation included the Wechsler Intelligence Scale for Children (WISC-IV) (11), Social Responsiveness Scale (SRS) (12), and assessment according to DSM-5 criteria. The evaluation concluded that she did not meet the diagnostic criteria for ASD.

Despite this conclusion, longstanding developmental concerns—including attentional variability, sensory sensitivities, and social difficulties—remained insufficiently explained, which led the parents to independently seek a second multidisciplinary evaluation at the same hospital-based developmental clinic three months later.

### 2.2 Second multidisciplinary evaluation at the hospital-based developmental clinic

The second diagnostic process at the hospital-based developmental clinic began with a comprehensive neurological evaluation, including a detailed developmental interview that incorporated all previous assessments. The neurologist observed short-duration eye contact, a small yet expressive range of facial expressions, and a discrepancy between the limited reciprocity expressed in verbal discourse and the intact reciprocity displayed during mutual play. Difficulties with impulse control and delayed gratification further affected the quality of reciprocal engagement. The developmental history reviewed by the neurologist was based on parental report, including sensory sensitivities, intense interests, repetitive behaviors, and rigid behavior. However, these behaviors were not observed in the neurologist’s examination.

In parallel, the psychological evaluation revealed strong social motivation, emotional warmth, and reciprocal interaction, accompanied by impulsiveness and challenges with emotional regulation. The psychologist noted a discrepancy between the ADOS-2 classification that showed a low level of autism related symptoms, and the Social Communication Questionnaire (SCQ) completed by the parents, in which her score exceeded the cut off for ASD. Detailed assessment scores and behavioral indicators are presented in **Table 1**.

**Table 1:** Summary of individual neurological and psychological assessments at the hospital-based developmental clinic.

Assessment Tool	Measure/Domain	Score	Interpretation
**ADOS-2 Module 3**	Total Score	5	Below the ADOS-2 threshold for ASD classification. Comparison score: 3 Indicates a low level of autism spectrum-related behaviors
**SCQ – Social Communication Questionnaire**	Total Score	19	Clinical cutoff is 15; the score exceeds the cutoff
**ABAS-II – Adaptive Behavior Assessment**	General Adaptive Composite	55	Very low range
	Conceptual Domain	63	Very low range
	Social Domain	66	Very low range
	Practical Domain	52	Very low range

ADOS-2, *Autism Diagnostic Observation Schedule, Second Edition*; a semi-structured observational instrument used for the assessment of Autism Spectrum Disorder. Module 3 is designed for children and adolescents with fluent speech, up to approximately age 15.

SCQ, *Social Communication Questionnaire*; a parent-report questionnaire assessing social communication skills and behaviors associated with Autism Spectrum Disorder.

ABAS-II, *Adaptive Behavior Assessment System, Second Edition*; a parent-report measure assessing adaptive functioning across conceptual, social, and practical domains.

The assessment process also included structured and open-ended parental questionnaires and administration of the CARS2-ST, which yielded a raw score of 20.5, indicating “minimal-to-no symptoms of ASD.” The CARS2-ST Version was selected because it is recommended for children over age 6 with below-average cognitive functioning and/or notable communication impairments, whereas the CARS2-HF is intended for verbally fluent individuals with average cognitive abilities. Despite this quantitative result, parental developmental history continued to include behaviors aligning with DSM-5 Criterion B, which refers to restricted and repetitive patterns of behavior, interests, or activities, such as sensory sensitivities, rigid adherence to routines, repetitive behaviors, or highly circumscribed interests. These features were reported consistently by the parents across assessments, although they were not directly observed during structured clinical encounters. At the same time, the neurologist’s clinical impression emphasized limited reciprocity in verbal contexts and subtle nonverbal communication difficulties.

These inconsistencies between quantitative testing, parental reports, and clinical observations resulted in diagnostic ambiguity. A multidisciplinary team discussion reflected divergent impressions—the neurologist suspected ASD, whereas the psychologist emphasized adaptive social motivation and reciprocity inconsistent with ASD. The team therefore recommended naturalistic peer-group observation to clarify the diagnostic picture.

### 2.3 Naturalistic peer group observations

Given the diagnostic ambiguity described above, and in light of known limitations of standardized ASD instruments in complex developmental profiles, the clinic employs a structured peer-group diagnostic observation technique that has been used in our hospital for over a decade (6). This approach was developed to support differential diagnosis in cases where individual assessments yield inconclusive or conflicting findings. As described in an article published in *Harefuah* in July 2025, and currently undergoing a formal standardization process, referral to the peer-group observation is indicated when there is disagreement between two independent clinicians (a child neurologist or psychiatrist and a psychologist) regarding whether the child meet criteria for ASD, or when a definitive diagnosis cannot be reached in the presence of suspected additional developmental or emotional complexity (6). Each diagnostic peer group consists of four to six children of mixed gender within a two-year age range. The observation protocol includes six weekly 50-minute sessions, co-facilitated by two clinicians who were not involved in the child’s prior individual assessments and who have formal training in ASD diagnostic tools. This structure is designed to enable repeated, naturalistic observation of spontaneous social communication and peer interaction over time.

Each session followed a consistent structure. Sessions opened with a brief 5–10-minute sharing round, followed by a short decision-making phase in which the group selected one of several activity formats, including conversation, collaborative storytelling or play, board games, art, construction, or physical activity. Approximately 30 minutes were devoted to the selected activity, and each session concluded with 5–10 minutes of individual reflection. Following each session, the facilitators systematically evaluated participants’ behaviors in relation to DSM-5 ASD criteria. After completion of the six-session sequence, observations were integrated across sessions to generate a comprehensive diagnostic formulation, which was subsequently presented and discussed at the multidisciplinary team meeting.

The child in this case participated in the peer-group diagnostic observation with age-matched peers. Across the six sessions, she generally arrived cheerful, although often late, and initially sought proximity to the facilitator during the first few minutes. She then integrated willingly into the group activity. Throughout the sessions, she frequently shared personal experiences and expressed emotions, demonstrating clear social interest and communicative intent. At the same time, her engagement was often self-focused, with a marked tendency to talk about her own experiences while showing reduced attentiveness to others. Importantly, her level of reciprocity fluctuated and appeared closely related to her degree of emotional and attentional regulation with which she arrived on a given day: when more regulated and attentive, her reciprocal engagement with peers increased noticeably. Her nonverbal communication was consistently intact. The child formed pleasant but relatively superficial connections with group members, particularly with the boys and with the facilitator, and her activity level appeared to align more closely with the interests of the boys in the group. Across all six sessions, attentional difficulties and hyperactivity were evident and interfered with her ability to sustain engagement and to deepen peer relationships over time. No restricted or repetitive behaviors were observed, nor were any sensory regulation difficulties noted. Adult support was required to maintain focus and manage frustration; however, the overall quality of social interaction fell within normative expectations for a child with developmental and attentional vulnerabilities.

### 2.4 Follow-up team discussion and final diagnostic impression

A second multidisciplinary meeting was held following the peer observation procedure. In addition to reviewing the observational findings, the team explicitly addressed the clinical formulation underlying the child’s repeated referrals for autism assessment. The formulation took into account both systemic and contextual factors, including the structure of the Israeli diagnostic system, which requires independent evaluations by a physician and a psychologist, as well as the fact that the family sought assessments from multiple professionals who were not in direct communication with one another.

The team further considered how the developmental history reported by the parents—emphasizing rigidity in play, restricted and intense interests, sensory sensitivities, and repetitive behaviors—had consistently directed clinical attention toward autism spectrum disorder. In the Israeli clinical and educational context, where an ASD diagnosis carries high salience and is widely recognized by families and professionals alike, these features were repeatedly interpreted within an autism framework, despite emerging evidence that warranted alternative explanations.

Based on the child’s demonstrated social-emotional reciprocity, use of nonverbal communicative behaviors, and ability to develop and maintain relationships with peers, the team concluded that she did not meet the DSM-5 criteria for autism spectrum disorder. Instead, the child’s difficulties were understood as reflecting below-average cognitive functioning, accompanied by Attention Deficit Hyperactivity Disorder and emotional immaturity. The team emphasized her social motivation, communicative intent, and emotional attunement, which weighed against an ASD diagnosis. This conclusion was informed by clinical observations from the structured group interaction assessment, summarized in **Table 2**. A final conference was held with the parents, during which the formulation and diagnostic conclusions were presented, alternative interpretations were discussed, and recommendations for ongoing support were jointly considered. The parents expressed agreement with the team’s conclusions and reported that the child was making meaningful progress at school, appeared emotionally settled, and was functioning well in her current educational setting. They noted that the formulation resonated with their own observations of the child’s strengths and challenges and welcomed the focus on addressing her attentional, emotional, and adaptive needs rather than pursuing an autism-specific intervention framework.

**Table 2:** Clinical observation summary—group interaction assessment.

Domain	Findings	Interpretation
**General Impression**	Participated consistently and actively; initiated interaction slightly late but joined shared activities and responded emotionally to the situation.	Demonstrates capacity for emotional involvement and shared play; engaged well despite some hesitation.
**Emotional-Social Reciprocity**	Initially hesitant, sat near caregiver; gradually integrated and shared positive affect. Needed encouragement to join group activities.	Some difficulty with spontaneous social initiative, but ability to participate with support.
**Nonverbal Communication**	Had eye contact and facial expressions.	No deficits observed.
**Peer Relationships**	Played parallel at first, then joined shared play with peers; accepted touch and interactions; at times-initiated interaction herself.	Shows social curiosity and engagement.
**Restricted or Repetitive Behavior**	None observed.	No evidence of stereotypic or restricted interests or behaviors.
**Summary/DSM-5 Impression**	Group interaction behavior did **not** support a diagnosis of Autism Spectrum Disorder (ASD).	Falls outside ASD criteria per DSM-5 based on this observation.

Overall clinical impression refers to the cumulative judgment of the examiners based on the child’s behavior across six diagnostic observation sessions.DSM-5 Criteria A1–A3 and Criterion B refer to the diagnostic criteria for Autism Spectrum Disorder as defined in the Diagnostic and Statistical Manual of Mental Disorders, Fifth Edition.Overall ASD judgment indicates the final clinical determination regarding the presence or absence of Autism Spectrum Disorder.

### 2.5 Summary of clinical findings

Summary tables of findings are presented in the Appendix:

Table 1 Summary of Individual Neurological and Psychological Assessments at the Hospital-based Developmental Clinic.

Table 2 Clinical Observation Summary—Group Interaction Assessment.

## 3 Discussion

This case underscores the diagnostic complexity involved in differentiating between various sources of social communication difficulties, as well as in distinguishing autism spectrum disorder (ASD) from other neurodevelopmental conditions (6). Children with developmental challenges often present with social and communication difficulties, which may arise from a range of underlying factors, including general developmental delays (13).

Standardized tools such as ADOS-2, CARS2-ST, and screening questionnaires, are valuable for offering a consistent framework for evaluating communicative behaviors across clinicians. However, their primary limitation lies in their inability to account for the underlying source of the observed behaviors (14, 15). Moreover, the scoring and interpretation of these tools relies on clinical judgment and are therefore inherently subjective (16–18).

This case highlights several key principles in the diagnostic evaluation of young children with complex developmental presentations. First, when multiple professionals are involved, effective interdisciplinary communication is essential to ensure that findings are integrated into a coherent diagnostic formulation rather than remaining fragmented across settings. Second, the case illustrates the limitations of relying solely on standardized or quantitative tools in the assessment of social communication. Such tools must be complemented by qualitative, clinically informed observations.

Third, extended naturalistic peer-group observation proved invaluable, offering insights into the child’s social communication abilities that were not evident in individual assessments with adult examiners. These observations provided a more ecologically valid understanding of her reciprocal interactions, emotional expression, and adaptive behavior within a real-world social context. Finally, the case highlights the importance of conveying clinical impressions and diagnostic conclusions to parents in a clear, accessible, and empathic manner, often across multiple conversations, so that they can fully understand the nature of their child’s strengths and difficulties and the rationale behind the diagnostic outcome.

## 4 Conclusion

The naturalistic peer-group observations reflected a strong desire for communication, intact nonverbal communication skills, reciprocity, and the ability to form and maintain interpersonal relationships with peers, subject to her attention regulation and ADHD symptoms. While the current findings highlight the clinical value of naturalistic peer-group observations, it should be noted that the peer-group observation technique is currently undergoing a process of standardization and quantitative validation. Ongoing research aims to establish structured scoring procedures and comparative benchmarks to differentiate between autistic and non-autistic children, thereby enhancing the method’s diagnostic validity and clinical applicability.

This case highlights the diagnostic challenges in assessing autism spectrum disorder in a young child presenting with a range of developmental difficulties, the importance of sustained multidisciplinary diagnostic teamwork, and the necessity of communicating the team’s conclusions and treatment recommendations to parents in a clear, consistent, and effective manner.

## Data Availability

The datasets presented in this article are not readily available due to ethical restrictions imposed by the institutional Helsinki Committee and medical confidentiality requirements. The data are anonymized, and access is restricted to the principal investigator.

## References

[1] American Psychiatric Association . Diagnostic and statistical manual of mental disorders. Fifth Edition. Arlington, VA: American Psychiatric Association (2013). doi: 10.1176/appi.books.9780890425596

[2] Lord C Rutter M DiLavore PC Risi S Gotham K Bishop SL . Autism diagnostic observation schedule, second edition (ADOS-2). Torrance, CA: Western Psychological Services (2012).

[3] Cook J Hull L Mandy W . Improving diagnostic procedures in autism for girls and women: A narrative review. NDT. (2024) 20:505–14. Available online at: https://www.dovepress.com/improving-diagnostic-procedures-in-autism-for-girls-and-women-a-narrat-peer-reviewed-fulltext-article-NDT (Accessed January 2, 2026)., PMID: 38469208 10.2147/NDT.S372723 PMC10926859

[4] Dworzynski K Ronald A Bolton P Happé F . How different are girls and boys above and below the diagnostic threshold for autism spectrum disorders? J Am Acad Child Adolesc Psychiatry. (2012) 51:788–97. doi: 10.1016/j.jaac.2012.05.018, PMID: 22840550

[5] Rynkiewicz A Zheng S Lacroix A . Special considerations for assessing and caring for autism in girls and women. Curr Opin Psychiatry. (2024) 37:71–7. doi: 10.1097/YCO.0000000000000913, PMID: 38085884

[6] Nagar-Shimoni H Gindi S Zilbershot Fink E Ben Shabbat Seri M Levy E Hutter-Beeri D . Autistic spectrum disorder (ASD): peer-group observation technique – three case reports. Harefuah. (2025) 164:412–7. 40704865

[7] Boyd BA Conroy MA Asmus J McKenney E . Direct observation of peer-related social interaction: outcomes for young children with autism spectrum disorders. Exceptionality. (2011) 19:94–108. doi: 10.1080/09362835.2011.565724

[8] Gilmore S Frederick LK Santillan L Locke J . The games they play: Observations of children with autism spectrum disorder on the school playground. Autism. (2019) 23:1343–53. doi: 10.1177/1362361318811987, PMID: 30413135 PMC6509020

[9] Bauminger-Zviely N Shefer A . Naturalistic evaluation of preschoolers’ spontaneous interactions: The Autism Peer Interaction Observation Scale. Autism. (2021) 25:1520–35. doi: 10.1177/1362361321989919, PMID: 33626914 PMC8323330

[10] Wechsler D . Wechsler preschool and primary scale of intelligence – third edition (WPPSI-III). an antonio. TX: The Psychological Corporation (2002).

[11] Wechsler D . Wechsler intelligence scale for children – fourth edition (WISC-IV). San Antonio, TX: The Psychological Corporation (2003).

[12] Constantino JN Gruber CP . Social responsiveness scale (SRS). Los Angeles, CA: Western Psychological Services (2005).

[13] Defresne P Mottron L . Clinical situations in which the diagnosis of autism is debata ble: an analysis and recommendations. Can J Psychiatry. (2022) 67:331–5. doi: 10.1177/07067437211041469, PMID: 34482753 PMC9065488

[14] Evers K Maljaars J Carrington SJ Carter AS Happé F Steyaert J . How well are DSM-5 diagnostic criteria for ASD represented in standardized diagnostic instruments? Eur Child Adolesc Psychiatry. (2021) 30:75–87. doi: 10.1007/s00787-020-01481-z, PMID: 32076870

[15] Colombi C Fish A Ghaziuddin M . Utility of the ADOS-2 in children with psychiatric disorders. Eur Child Adolesc Psychiatry. (2020) 29:989–92. doi: 10.1007/s00787-019-01411-8, PMID: 31587085

[16] Kamp-Becker I Albertowski K Becker J Ghahreman M Langmann A Mingebach T . Diagnostic accuracy of the ADOS and ADOS-2 in clinical practice. Eur Child Adolesc Psychiatry. (2018) 27:1193–207. doi: 10.1007/s00787-018-1143-y, PMID: 29560529

[17] Frigaux A Evrard R Lighezzolo-Alnot J . L’ADI-R et l’ADOS face au diagnostic différentiel des troubles du spectre autistique : intérêts, limites et ouvertures. L’Encéphale. (2019) 45:441–8. Available online at: https://linkinghub.elsevier.com/retrieve/pii/S0013700619302362 (Accessed January 2, 2026). 10.1016/j.encep.2019.07.002 31495549

[18] Greene RK Vasile I Bradbury KR Olsen A Duvall SW . Autism Diagnostic Observation Schedule (ADOS-2) elevations in a clinical sample of children and adolescents who do not have autism: Phenotypic profiles of false positives. Clin Neuropsychologist. (2022) 36:943–59. doi: 10.1080/13854046.2021.1942220, PMID: 34294006

